# Preparation, Characterization, and Antiangiogenic Evaluation of a Novel 5-Fluorouracil Derivative Solid Lipid Nanoparticle with a Hen’s Egg Chorioallantoic Membrane Assay and Wound Healing Response in HaCaT Keratinocytes

**DOI:** 10.1021/acsomega.4c00635

**Published:** 2024-03-25

**Authors:** Çinel Köksal Karayildirim

**Affiliations:** Faculty of Science, Department of Biology, Ege University, Izmir 35100, Turkey

## Abstract

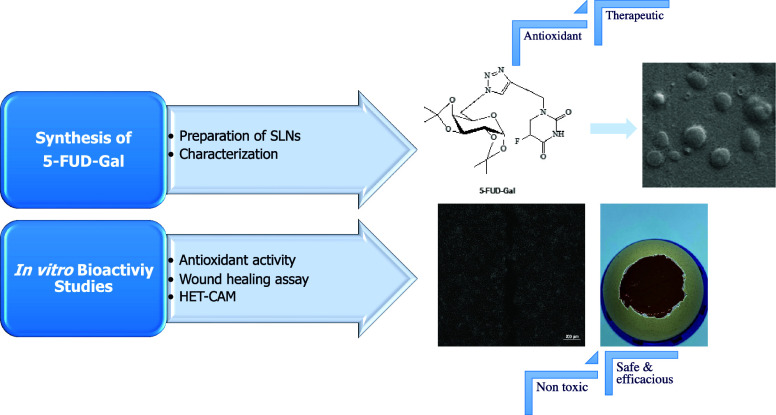

5-Fluorouracil is a heterocyclic aromatic organic compound, and it is commonly used as a chemotherapeutic agent in many cancers. The present goal is to analyze and characterize the physicochemical and biological properties of a new therapeutic formulation of 5-FUD-Gal under simulated chronic wound and oxidative stress conditions. After synthesis of a new 5-fluorouracil derivative, preparation and characterization of the formulation were carried out. The antiangiogenic effect, wound healing, and oxidative stress responses were conducted with a HET-CAM assay and *in vitro* cell culture technique. The results initially demonstrated that 5-FUD-Gal synthesized by a series of reactions and the SLN formulation were prepared successfully. A strong cell protective effect above 98% cell viability was detected at 20 μM at 48 h. The wound closure of the HaCaT scratch assay was calculated to be 90.12 and 98.98% at 10 and 20 μM concentrations, respectively, at 48 h. Moreover, the strongest effect of 5-FUD-Gal-F was observed at 20 μM concentration on chicken embryos. This study provides novel insights that a new derivative of semisynthetic 5-FUD-Gal-F can be further evaluated as a therapeutic chemical compound in cancer disease.

## 1 Introduction

5-Fluorouracil (5-FU), a pyrimidine antimetabolite used as a highly toxic chemotherapeutic agent, has been widely used for over 50 years in the treatment of many types of solid tumors such as colon, skin, and breast cancers.^1,2^ 5-FU is an analogue of uracil that has a fluorine atom at the C5 position instead of hydrogen, and with regard to its molecular mechanism of interaction with cancer calls, it is not strongly tumor selective. However, the main problem with 5-FU is that it causes many toxic effects in the hepatic, neurovascular, and gastrointestinal systems. Therefore, we need to better understand why structural modifications of 5-FU have been made. There have been many studies done on 5-FU where 5-FU has been bound to peptides, phospholipids, amino acids, and polymers reported as a prototype.^3,4^ Derivatization at the 1 and/or 3 positions of 5-FU resulted in improved biological attributes, such as increased activity, selectivity, metabolic stability, and absorption and decreased toxicity. Based on this strategy, a new derivative of 5-FU containing nucleobase and sugar was synthesized for higher antiangiogenic bioactivity to cancers. It has been demonstrated that the synthesis of a novel derivative of 5-FU has an advantage over the receptor-mediated uptake of 5-FU via utilization of these elevated receptors on cancer cells.^5,6^

Recently, new drug delivery systems have been reported as the most promising approach in the fight against cancers.^7,8^ A variety of drug delivery systems developed for incorporating anticancer agents include solid lipid nanoparticles (SLNs) as well as macromolecular nanoparticles and liposomes.^9,10^ SLNs are made up of a solid central lipid component and a surfactant that surrounds it and helps dispersion of lipophilic components in aqueous solutions. As compared to other drug delivery systems, these systems have many advantages, including the ability to combine hydrophilic and lipophilic drugs, the absence of toxicity, and the ability to scale up production.^11,12^

Chemotherapy-resistant tumors show increased metastasis and proliferation that are associated with angiogenesis. In particular, angiogenesis inhibitors are expected to yield tumor growth inhibition rather than tumor shrinkage. It is well-known that angiogenesis is a key role in the wound healing process after a physical injury or disruption of tissue regeneration.^13^ Targeting angiogenesis can be an effective approach to preventing the development of new blood vessels.

In this study, it was proposed that a new derivative of 5-FU having sugar in pyranose form and nucleobase could possess great antiangiogenic potential, which is related with wound healing activity by using *in vitro* cell culture techniques. In this regard, SLNs were prepared to enhance therapeutic efficacy and avoid adverse effects of conventional chemotherapy in the fight against tumors. This formulation included a new derivative of semisynthetic 5-FU components to shape the antiangiogenic roles targeting the tumor microenvironment. The study was aimed to emphasize the significance of modulating the tumor microenvironment and for the first time illustrate the potential of a novel derivative of 5-FU pharmacological targeting in cancer therapy. Moreover, 5-FU and its derivatives are generally used for cancer therapy, but here, for the first time, the wound healing effects were investigated.

## 2 Materials and Methods

### 2.1 Materials

All chemicals were of analytical grade and were supplied from Merck. Cell culture materials were purchased from Sigma-Aldrich. Lutrol-F68 was provided by BASF.

### 2.2 Methods

#### 2.2.1 Synthesis of 1-[{1′-(6″-Deoxy-1″,2”:3″,4″-di-*O*-isopropylidene-α-D-galactopyranos-6″-yl)-1′*H*-1′,2′,3′-triazol-4′-yl}methyl] 5-fluorouracil (5-FUD-Gal)

**Figure 1:**
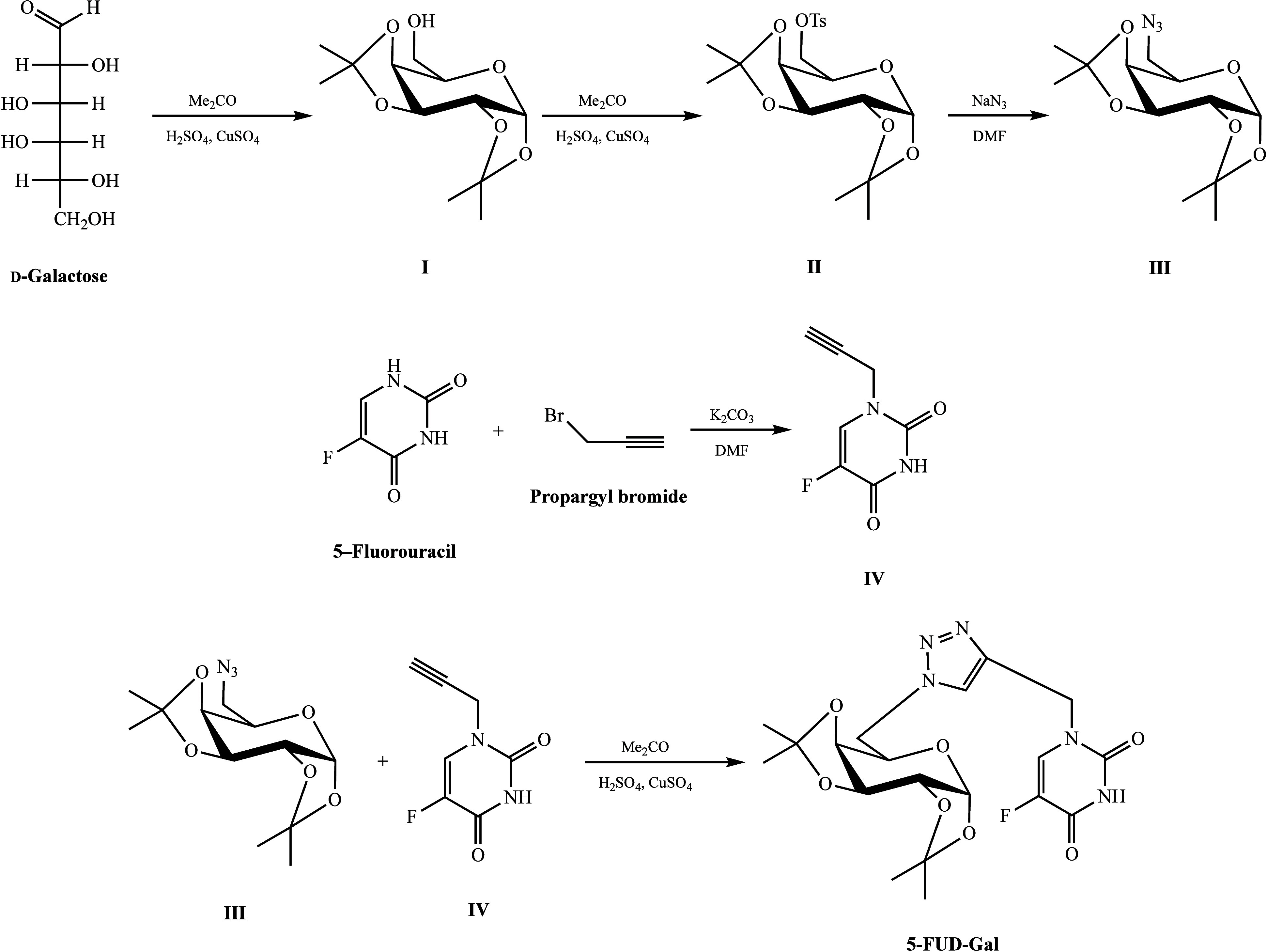
Synthetic route of 5-FUD-Gal.

To synthesize 5-FUD-Gal, azido galactopyranose (**III**) was synthesized by a series of reactions, protection of the hydroxyl group on the 2,3,4,5 positions, tosylation of the hydroxyl group on the 6 position, and substitution of the tosyl with the azido group. Then, *N*-1-propargyl 5-fluorouracil was synthesized from 5-fluorouracil and propargyl bromide (**IV**). Finally, 5-FUD-Gal was synthesized from azido galactopyranose (**III**) and *N*-1-propargyl 5-fluorouracil (**IV**) via copper-catalyzed azide–alkyne cycloaddition click reaction (Figure 1).^14^

#### 2.2.2 Preparation of 5-FUD-Gal-Loaded SLNs

The lipid phase (40 mmol L^–1^) of the SLN formulation was prepared by molten stearic acid and lecithin mixtures at molar ratios of 8:2 and 6:4 in acetone solution (5 mL). X, at a drug-to-lipid mass ratio of 1:5, was put into distilled water at 60 °C to form a water/oil/water (W/O/W) emulsion as a hydration medium in formulations. The W/O/W emulsion was then added to 10 mL of cold water at 4 °C under 500 W and 20 kHz in alternating 20 s cycles for 15 min by using a Vibracell tip sonicator, forming SLN. The obtained SLN formulation was filtered through polycarbonate membrane filter units (0.22 μm pore size, Millipore) to select for particles <220 nm. 5-FUD SLN formulation with a 5-FUD-to-lipid mass ratio of 1:5 was coded as X-SLN.

#### 2.2.3 Characterization Studies

##### 2.2.3.1 Particle Size and Zeta Potential of Formulations

The mean particle size, polydispersity index (PDI) value, and zeta potential of the formulations were determined by a Zetasizer (Malvern, UK). The mean particle size and PDI values of the formulations were measured with the dynamic light scattering method.

The zeta potential of samples was measured by a zeta cell cuvette. The Helmholtz–Smoluchowski equation was used to evaluate the zeta potential of the formulations from the electrophoretic mobility under an electrical field of 40 V/cm. All measurements were performed at least five times at 25 ± 2 °C.

##### 2.2.3.2 Scanning Electron Microscopy of Formulations

Scanning electron microscopy (SEM) (Thermo Scientific Apreo S brand) was used to evaluate size and surface properties of developed formulations. The formulations were coated with 80% gold and 20% palladium in 7 nm thickness under 5 × 10^–4^ mBar vacuum administration. A magnification range of 50.000× was applied to all samples above 5 kV voltage conditions.

#### 2.2.4 Stability Study

The stability of formulations was evaluated under 5 °C, 25 °C and 60% relative humidity (RH), 40 ± 5 °C and 75 ± 5% (RH) for 3 months. The particle size, PDI, zeta potential, and formulation appearance by visual inspection were observed, and statistical analyses for all parameters were carried out.

#### 2.2.5 *In Vitro* Antioxidant Activity Test in Vero Cells

##### 2.2.5.1 Cell culture

The *Cercopithecus aethiops* kidney tissue and Vero adherent fibroblast cells (ATCC CCL-81.5) were used to evaluate the antioxidant capacity of the 5-FUD-Gal formulation.^15^ Vero cells were cultured for 3 days with Dulbecco’s modified Eagle’s medium in a 5% CO_2_ atmosphere at 36 °C. After incubation time, cell pellets were prepared at a concentration of 1.5 × 10^5^ mL in 96-well microplates. The study was assessed in the absence of an antibiotic solution to avoid false-positive results.

##### 2.2.5.2 Determination of the Protective Effects of 5-FUD-Gal Formulation against H_2_O_2_-Induced Cytotoxicity in Vero Cells

Vero cells were used to determine the protective effects of 5-FUD-Gal formulation against H_2_O_2_-induced cytotoxicity. When the cells reached 95–100% confluence, 5-FUD-Gal formulations in 5% of DMSO solutions at various concentrations (5, 10, and 20 μM) were added into 96-well plates and incubated for 1 h at 37 °C. DMSO was added to the medium to dissolve the MTT formazan crystals. The absorbance was measured at 570 nm by using a microplate reader. Cell viability percentage was determined as the mean of cell survival in three experiments.^16^

#### 2.2.6 *In Vitro* Scratch Wound Healing Assay on HaCaT Cells

The evaluation of the *in vitro* scratch wound healing assay was performed on human epidermal keratinocyte cells (HaCaT, ATCC PCS-200-011). The cells were at a seeding density of 5 × 10^5^ cells/mL in a DMEM medium with 10% FBS, 1% penicillin-streptomycin applied to nearly confluent cell monolayers at 37 °C under a 5% CO_2_ atmosphere and constant humidity. The mechanical artificial gap of the scratch area was constituted in the confluent HaCaT cell monolayer (5 × 10^4^ cells/cm^2^) with polypropylene pipet chips. Different concentrations of 5-FUD-Gal formulation solutions in DMSO (5, 10, and 20 μM) were then incubated with the cells for 48 h. The control cells were also scratched and maintained in the medium with 1% FBS. The width of the gap was measured, and the percentage of wound healing results was evaluated according to the cells’ migration into the scratched region after 5-FUD-Gal-F exposure by using ImageJ software.^17^ The untreated cells were defined as a control group (100% live cell). The cell migration experiment was conducted at least twice using three wells for each stimulating condition.

#### 2.2.7 Evaluation of the Antiangiogenic Effect of 5-FUD-Gal Formulation with the Hen’s Egg Chorioallantoic Membrane Assay

##### 2.2.7.1 Preparation of Fertilized Eggs

Fertilized White Leghorn 50–60 g hen’s eggs were kept at a horizontal position at 36.0 °C and 65% relative humidity in an automatically turning egg incubator on day zero. Eggs were rotated for nine days to prevent the attachment of the embryo to one side of the shell and away from the CAM. On the ninth day, fertilized eggs were checked by a lamp, and the shell was marked on the line of the airspace to prepare for the assay. The 3–5 cm diameter of the shell was removed, and the membrane was moistened with 2 mL of a 0.9% saline solution. A Parafilm was used to cover the CAM area, and the eggs were incubated at 36.0 °C for 70 h.

##### 2.2.7.2 HET-CAM Assay

The pellets consisted of 10 μL of gelled 3% w/v agarose applied directly onto the CAM for topical application. At least 15 eggs per condition were used. The different concentrations of 5-FUD-Gal formulation in 5% DMSO were prepared (5, 10, and 20 μM). Negative and positive control groups consisted of 0.9% saline and suramine and 50 μM thalidomide, respectively. After the administration of all the groups, the eggs were incubated at 36.0 °C for 24 h, and the experiments were performed twice. Vascular parameters such as lysis, hemorrhaging, and/or coagulation were evaluated according to a score system.^18^

#### 2.2.8 Statistical Analysis

The data was analyzed using GraphPad Instat software (San Diego, USA). The experimental data was expressed as mean ± SEM. One-way ANOVA was used to compare the mean values of treated and control groups in SPSS 25.0. In all studies, acceptable significance was observed, and the *p* value was <0.05.

## 3 Results

### 3.1 Synthesis of 1-[{1′-(6″-Deoxy-1″,2”:3″,4″-di-*O*-isopropylidene-α-D-galactopyranos-6″-yl)-1′*H*-1′,2′,3′-triazol-4′-yl}methyl]5-fluorouracil (5-FUD-Gal)

The structural elucidation of azido galactopyranose (**III**), *N*-1-propargyl 5-fluorouracil (**IV**), and 5-FUD-Gal was examined by FT-IR and NMR spectra, and all spectroscopic data are compatible with our previous study.^14^

### 3.2 Preparation of Formulations

5-FUD-Gal SLNs were prepared by an emulsification method. There is a lipid tail and core parts in formulations, and 5-FUD-Gal is connected with the tail and core of the lipid phase. Also, 5-FUD-Gal is associated with the lecithin monolayer. Van der Walls forces can affect these connections by the lipid portion of the lipid-5-FUD-Gal lecithin molecule and association with the lipid core of the SLN.

Particle sizes of all formulations were found to be below 100 nm (Table 1). As expected, the particle sizes of 5-FUD-Gal SLN formulation were found to be higher than empty SLN due to their drug content. The PDI values show the homogeneity of particle size distribution in SLNs. A PDI value of 0.2 is desired for monodispersity of formulations.^2^ Herein, PDI values are under 0.2, expressing the homogeneity of the size distribution of all SLN formulations. The PDI represents the width of the particle sizes, where values <0.3 represent a very narrow size distribution and ≤0.5 represent only a narrow size distribution, which is absolutely acceptable for drug delivery systems.

**Table 1:** Characterization Properties of SLN Formulations

**formulation**	**mean particle size (nm)**	**PDI**	**zeta potential (mV) ± SD**
SLN	84 ± 3.12	0.5 ± 0.1	–24 ± 3.8
5-FUD-Gal SLN	95 ± 2.32	0.4 ± 0.11	–28 ± 2.2

**Figure 2:**
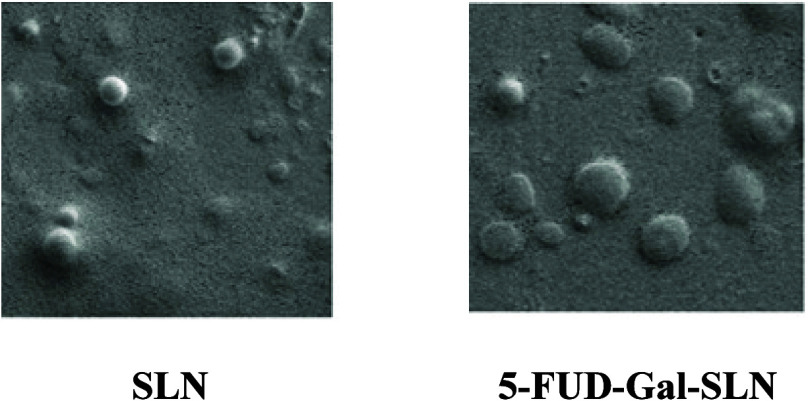
SEM images of SLN formulation.

Hence, SLN formulations were found as polydisperse samples. Furthermore, SEM experiments confirmed that the morphology of SLNs was almost spherical in nature (Figure 2).

Zeta potential is an important parameter in terms of stability because it shows the electrokinetic property and aggregation possibility of lipid vesicles. The phospholipid type, stearic acid, and hydrophilic phase in the content of SLNs have critical roles in the zeta potential value of vesicles.^19^ Zeta potential measurements showed that the SLN formulations were negatively charged. Interestingly, most of the FDA-approved lipid drug formulations are negatively charged lipid particulate systems.^20^

The stability of formulations is shown in Table 2, and the results proved that the stability of formulations did not change during storage conditions and time intervals.

**Table 2:** Stability Results of SLN Formulations

	**initial values**			**3 months values**								
	**25 °C**			**5 °C**			**25 °C**			**40 °C**		
**formulations**	**mean particle size nm**	**PDI**	**zeta potential (mV) ± SD**	**mean particle size nm**	**PDI**	**zeta potential (mV) ± SD**	**mean particle size nm**	**PDI**	**zeta potential (mV) ± SD**	**mean particle size nm**	**PDI**	**zeta potential (mV) ± SD**
SLN	84 ± 3.12	0.5 ± 0.10	–24 ± 3.8	89 ± 2.34	0.4 ± 0.08	–25 ± 2.88	85 ± 5.67	0.4 ± 0.10	–25 ± 2.6	88 ± 3.8	0.3 ± 0.09	–26 ± 2.8
5-FUD-Gal-SLN	95 ± 2.32	0.4 ± 0.11	–28 ± 2.2	97 ± 1.08	0.5 ± 0.09	–29 ± 3.13	90.23 ± 4.5	0.3 ± 0.10	–29 ± 1.1	97 ± 1.1	0.4 ± 0.10	–27 ± 3.9

### 3.3 Determination of 5-FUD-Gal Formulation on H_2_O_2_-Induced Intracellular ROS Scavenging Activity

**Figure 3:**
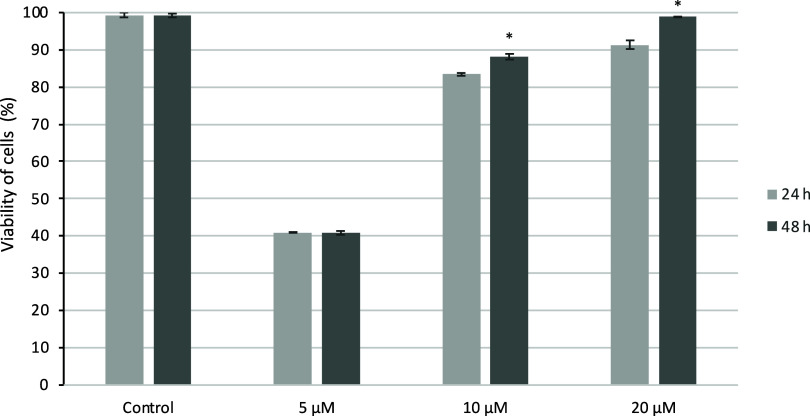
Cell-based antioxidant activity of 5-FUD-Gal formulation on cell viability of H_2_O_2_-induced Vero cells. Statistical evaluation was performed to compare the experimental groups and corresponding control groups, *p < 0.05.

The 5-FUD-Gal formulation pretreatment of cells resulted in the inhibition of H_2_O_2_-induced cell damage, and cell protective effects such as a decreased ROS level and increased cell viability against oxidative stress were also demonstrated. This study revealed that 5-FUD-Gal-F exhibits dose-dependent protective effects since the cell viability ratio changed with different concentrations ranging from 5 to 20 μM. It was shown that the scavenging activity of 5-FUD-Gal-F on reactive oxygen species and the viability of Vero cells decreased significantly when caused by H_2_O_2_-induced oxidative stress at 10 and 20 μM concentration levels. The strong cell protective effect above 98% cell viability was detected in the highest concentration of 5-FUD-Gal-F compared to the untreated cells at 48 h. Additionally, 5-FUD-Gal-F induced cell viability above 88% at 10 μM at 48 h (Figure 3).

### 3.4 *In Vitro* Scratch Wound Healing Assay on HACAT cells

**Figure 4:**
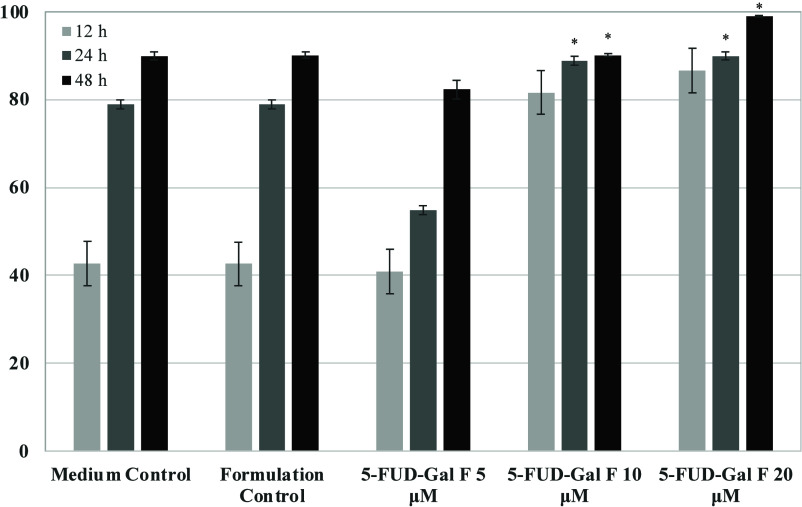
Effect of 5-FUD-Gal-F on HaCaT cell wound healing at 12, 24, and 48 h. The controls are represented by the wound healing of medium and formulation controls. *p* < 0.05 compared with untreated culture (medium control).

**Figure 5:**
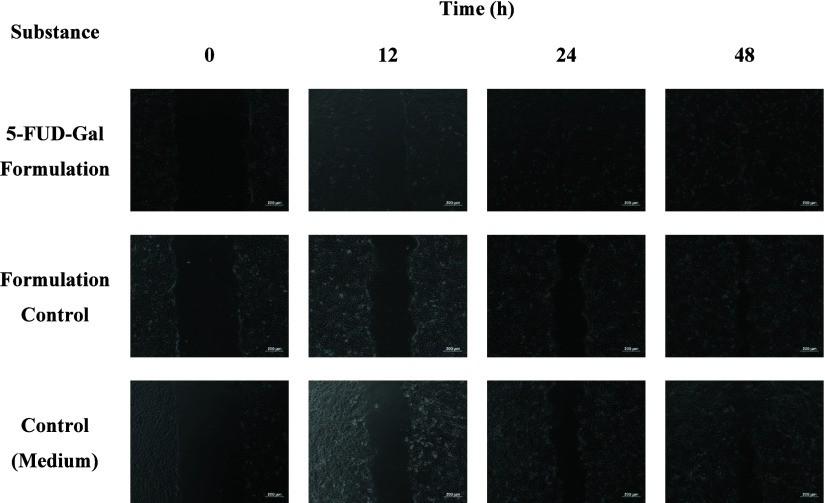
Measurement of cell migration in the *in vitro* scratch assay on the HACAT cell layer subjected to scratch and treated with the medium control, 5-FUD-Gal formulation (20 μM), and formulation control. Images captured at 10× magnification using a microscope (AxioCam, Germany), at times 0, 12, 24, and 48 h after incubation. *p* < 0.05 compared with untreated culture (medium control).

An *in vitro* scratch test was used to determine the ability of 5-FUD-Gal-F in wound healing on 95%–100% confluence HACAT cells. The measurement of migrated cells was conducted by assessing the closure of the cell-free area. The angio-therapeutical effect of 5-FUD-Gal-F on cell damage, proliferation, and quantification of wound closure was calculated. The evaluation of the wound area reduction depended on the concentration level and time. The initial width of scratched areas was calculated as 42.67 ± 2.52% for untreated HaCaT control cells at 12 h when 5-FUD-Gal-F was at 20 μM assessed as 86.59 ± 1.50%. It was shown that 48 h of 5-FUD-Gal-F exposure significantly induced cell migration. The wound closure of the HaCaT scratch assay was calculated as 90.12 ± 0.29% and 98.98 ± 0.13% at 10 and 20 μM concentrations, respectively, at 48 h (*p* < 0.05) (Figures 4 and 5).

### 3.5 HET-CAM Test Results

The antiangiogenic effects of 5-FUD-Gal formulation were demonstrated in the HET-CAM assay. The strongest effect of 5-FUD-Gal-F was observed at a 20 μM concentration on chicken embryos (Table 3).

**Table 3:** Antiangiogenic Effects of Different Concentrations of 5-FUD-Gal Formulation and Control Groups

**samples**	**concentration pellet**	**score**	**antiangiogenic effect**
5-FUD-Gal-SLN	5 μM	0.58 ± 0.05	weak
	10 μM	1.82 ± 0.02	good
	20 μM	2.53 ± 0.03	very good
thalidomide	50 μM	1.14 ± 0.03	good
suramine	50 μM	0.47 ± 0.02	weak
saline solution	9%		none

**Figure 6:**
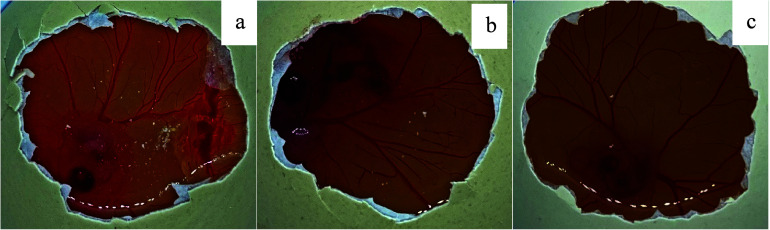
HET-CAM assay control groups: (a) strong damage capillary area on the CAM caused by 50 μM thalidomide; (b) weak antiangiogenic effect of treatment with 50 μM suramine; and (c) no antiangiogenic effect of 9% saline solution.

**Figure 7:**
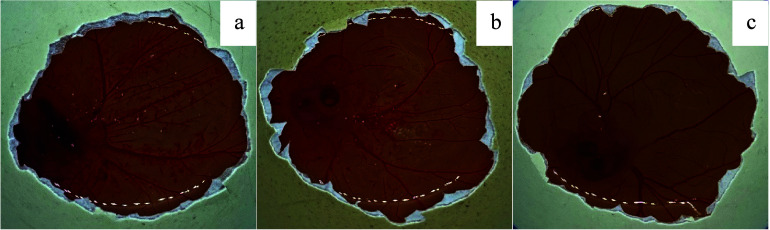
HET-CAM assay: induction of angiogenesis. (a) Strong antiangiogenic effect caused by 20 μM 5-FUD-Gal. (b) Good antiangiogenic effect caused by 10 μM 5-FUD-Gal. (c) Normal growth of blood capillaries on the CAM—no antiangiogeniceffect with 5 μM 5-FUD-Gal.

The hemorrhagic activity of 5-FUD-Gal-F with a 20 μM concentration was higher than thalidomide. Sumarine and 5-FUD-Gal-F revealed the same weak antiangiogenic efficiency at 5 μM (Figures 6 and 7).

## 4 Discussion

Today, cancer is one of the major public health problems in the world and continues to cause many deaths; the affected population is increasing day by day. Although many anticancer drugs have been discovered and developed for use in clinical treatment over the past few decades, most of the available drugs cause serious side effects and are nonselective to acquire drug resistance.^21^ Since tumor development and progression are highly dependent on angiogenesis, in recent years, the use of antiangiogenic therapy as an alternative treatment for cancer patients has attracted considerable interest from researchers.

In this study, it was demonstrated that current strategies and recent research in the production of valuable therapeutic compounds are important and highlight the semisynthetic antiangiogenic modulators as an emerging class of anticancer agents. Therefore, it is important to develop new and effective semisynthetic compounds to fight against diseases that are biologically active and nontoxic. A 5-FU derivate-compound that contains galactose and nucleobase groups was used as a pharmaceutical part. These two groups play an important role in that they have a higher affinity to cancer cells.^14^ Therefore, we thought that modifying 5FU in these groups could affect the cell-binding capacity of the newly designed compounds. The findings, similar to other previous reports, indicated that the semisynthetic antiangiogenic compounds have great potential to be a source of modern drugs to treat different types of cancers.^5^

Encapsulation of new molecules in drug delivery vehicles is a common strategy used in drug delivery to overcome the undesired properties of effective molecules. Based on these considerations, an SLN formulation containing 5-FUD-Gal was developed and evaluated with characterization and stability studies.

The particle size measurement, zeta potential measurement, and SEM imaging of the developed formulation were performed. Particle sizes of drug delivery systems play a critical role in their nonspecific accumulation in the tumor tissue by the enhanced permeability and retention (EPR) effect. For the EPR phenomenon, drug delivery systems should escape from the reticuloendothelial cells and remain for a longer time in the blood circulation. Nanoparticles averaging ∼100 nm have been reported to be the most suitable for extending the blood circulation half-life.^22^ Therefore, the developed SLN formulations were found to be appropriate in terms of particle size (Table 1) for remaining longer in circulation and nonspecifically targeting the tumor by the EPR effect. According to the literature, the PDI value should be under 0.2 to obtain a monodispersed formulation.^23^ Therefore, SLN formulations have midrange polydispersity, as described in Table 1.

The *in vitro* wound healing assay allows the quantitative evaluation of cell migration, which is a normal physiological process.^24^ Free-radical scavenging efficacy and inhibition of ROS are two effective ways of preventing cell damage via antioxidant compounds during the wound healing process.^25^ This process is mainly associated with induced angiogenesis, proliferation of fibroblasts, keratinocytes, and endothelial cells, expanded migration, and regulation of growth factors. This study showed that 5-FUD-Gal-F promoted cell migration and re-epithelialization and accelerated the initiation of wound healing in soft tissue. Evaluation of the wound healing performed in different concentrations of 5-FUD-Gal-F revealed that the quickest scratch zone closure was obtained in 10 and 20 μM concentrations. After 48 h, the wound width in 20 μM 5-FUD-Gal-F exposed cells was significantly smaller than that in untreated control cells. Results demonstrated that the 5-FUD-Gal-F formulation enhances wound healing, which means the angiogenesis process after treatment may play a key role in tissue remodeling after anticancer therapy.

The HET-CAM assay can be used for the evaluation of the toxic side effects of the test substance and stimulate the evaluation of newly formed vessels and proangiogenic growth factors for neovascularization. 5-FUD-Gal-F strongly induced vascularized granuloma in the CAM at the 10 and 20 μM/pellet concentrations. The strongest antiangiogenic effect was observed at a concentration of 5-FUD-Gal-F at 20 μM. The capillary formation was inhibited by 5-FUD-Gal-F in a dose-dependent manner without showing any rupture or toxicity to blood vessels. There are several scientific investigations that have been carried out on the antiangiogenic activity of semisynthetic compounds.^26,27^ It is known that angiogenesis is promoted by 5-fluorouracil in many types of cancers. However, the importance of this study is in the evaluation of a more effective and active novel semisynthetic target molecule with antiangiogenic activity. This evidence suggests that 5-FUD-Gal-F may be a remarkable target of antiangiogenic therapy.

Based on the results obtained from the *in vitro* studies, it is shown that the antiangiogenic impact of 5-FUD-Gal-F for cancer therapy would be greatly enhanced by the availability of a new, simple, and easy healing process because of a molecular structure similar to the pyrimidine dimers in DNA and RNA structures.^28^ The findings show the potential of the 5-FUD-Gal-F formulation as a semisynthetic compound that possesses strong antiangiogenic activity with a high wound healing effect.

## 5 Conclusions

Semisynthetic drug-biomolecules are important in the development of new pharmaceuticals. Accordingly, the purpose of the current study was to investigate the potential wound healing and antiangiogenic effects of 5-FUD-Gal formulation *in vitro*. Taken together, this is the first study where 5-FUD-Gal-F, a semisynthetic, could promote the healing of wounds and regulate angiogenesis. This study highlighted the biological effects of 5-FUD-Gal-F and its possible mechanisms that can substantiate advantageous benefits for the treatment of cancer.

## Notes

Author declares that this manuscript is original, has not been published before, and is not currently being considered for publication elsewhere.
